# Whole-genome sequence of the African Common Reed Frog (*Hyperolius viridiflavus viridiflavus*) from Ethiopia

**DOI:** 10.1093/g3journal/jkaf257

**Published:** 2025-10-27

**Authors:** Lucinda P Lawson, Sandra Goutte, H Christoph Liedtke

**Affiliations:** Center for Autoimmune Genomics and Etiology, Cincinnati Children's Hospital Medical Center, Cincinnati, OH 45229, United States; Division of Allergy and Immunology, Cincinnati Children's Hospital Medical Center, Cincinnati, OH 45229, United States; Biological Sciences, University of Cincinnati, Cincinnati, OH 45221, United States; Division of Science, New York University Abu Dhabi, PO Box: 129188, Saadiyat Island, Abu Dhabi, United Arab Emirates; Department of Evolution and Ecology, Doñana Biological Station (CSIC), 41092, La Cartuja Island, Seville, Spain

**Keywords:** genome assembly, Afrobatrachia, amphibian, species-complex

## Abstract

Amphibians of the Afrobatrachia clade represent a major component of sub-Saharan Africa's biodiversity, yet they remain underrepresented in genomic databases. Here, we present the first whole-genome assembly of the Common Reed Frog*, Hyperolius viridiflavus viridiflavus* Ahl, 1931 from Ethiopia, a member of the *H. viridiflavus* superspecies complex. The genome was sequenced using PacBio HiFi long-read technology and assembled de novo with HiFiasm, resulting in a 4.4 Gb assembly across 10,009 contigs with an N50 of 1.09 Mb. Genome completeness had a BUSCO score of 84.2%, with 29,809 annotated genes, including 27,983 protein-coding genes and 942 long non-coding RNAs. Despite a similar estimated size, scaffolding against the chromosome-level genome of *Hyperolius riggenbachi* Nieden, 1910 revealed low mapping coverage (0.0654 per base across 1 Mb windows), likely due to phylogenetic divergence (∼14 Mya) and high repeat content. The complete mitochondrial genome (23,453 bp) was also assembled and annotated, revealing structural differences from closely related species. Phylogenomic analyses using 416 single-copy BUSCO genes and mitochondrial 16S sequences confirmed the distinctiveness of *H. v. viridiflavus* within the Hyperoliidae. As only one other Afrobatrachian genome exceeds 50% completeness in public databases (*H. riggenbachi*), this genome expands the resources available for African frogs and supports future research in systematics and conservation. Further, when considering the complex taxonomy and evolutionary history of the *H. viridiflavus* superspecies complex, this genome can serve as a tool for species delimitation and conservation when compared to other species and subspecies within the clade.

## Introduction

The African Common Reed Frog, *Hyperolius viridiflavus*, represents a superspecies complex of ∼60 species and subspecies found throughout sub-Saharan Africa (Duméril and Bibron 1841; Schiøtz 1975; Channing and Howell 2006). Within this complex there are a number of named species and subspecies with uncertain taxonomic status or geographical boundaries (Schiøtz 1999; Wieczorek et al. 2001; Portik et al. 2019; Channing 2022). Understanding the evolution and diversification of this radiation first requires creating genomic resources, yet whole genome resources for amphibians in general are scarce (Kosch et al. 2024). Afrobatrachia, consisting of four families that are endemic to the African continent (Arthroleptidae, Brevicipitidae, Hemisotidae, and Hyperoliidae) have been entirely absent for whole genomes prior to 2024. In comparison to other anuran lineages, Afrobatrachia are therefore under-sampled despite representing more than half of the diversity of frogs on the African continent, leaving a large section of the amphibian tree-of-life without whole genome resources (Womack et al. 2022).

Here we provide the first whole-genome of a member of the *Hyperolius viridiflavus* superspecies complex. This sub-Saharan African superspecies complex is comprised of approximately 150 taxa representing recognized species and subspecies with unclear taxonomic standing and relationships (Schiøtz 1999; Portik et al. 2019; Channing 2022). Most taxa within the complex were originally described based on allopatric ranges and divergent coloration. Taxa within this complex are exceptionally brightly colored when compared to other members of the *Hyperolius* genus. Colors range from reds, greens, yellows, blacks, whites, browns, and more. Patterns include spots, splotches, stripes, and “immaculate” forms. For this genome, we sampled a *H. viridiflavus viridiflavus* specimen from Jimma, Ethiopia, with the correct coloration for and within the geographical range of *Hyperolius viridiflavus viridiflavus* (Schiøtz 1999; Channing 2022). By precisely establishing this specimen as a reference to which all other members of the *H. viridiflavus* superspecies complex can be aligned, the systematics and associated conservation implications can be ultimately determined.

**Fig. 1.**
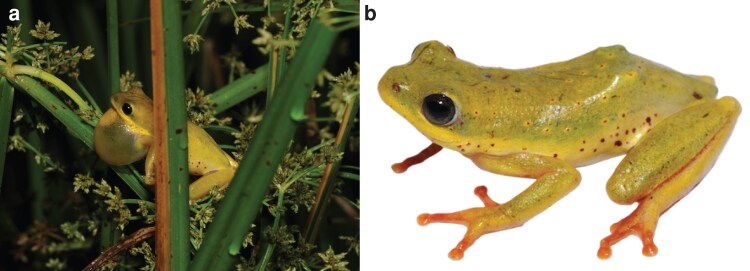
Photos of *Hyperolius viridiflavus viridiflavus* male near Jimma Ethiopia (SB243). a) photo *in situ* with gular sac extended. b) Reference photo. Photos from Sandra Goutte.

To assemble this genome, an adult male *Hyperolius viridiflavus viridiflavus* (field ID SB243, NCBI BioProject PRJNA1278718) was collected at night on 24 April 2018 by S. Goutte in a pond overgrown with high reeds in an old quarry Southeast of Jimma (7°32'06 N, 36°33'38 E; 2,216 m a.s.l.), Ethiopia (Fig. 1). This location is within the range of *Hyperolius viridiflavus viridiflavus* (Schiøtz 1999). Approximately 750 km separate this locality and the type locality of *Hyperolius viridiflavus* (Adwa, Ethiopia; Lefebvre 1851), but as no closer specimens of suitable quality for genomics (samples were tried from the type locality housed at the British Museum of Natural History and failed quality control), we consider this a suitable representation due to the established range in Western Ethiopia and the characteristic coloration of this subspecies. Though Jimma is nearer the type locality of *Hyperolius v. destefanii* (“Nargi”, Ethiopia, ∼300 km; Scortecci 1943), this alternate subspecies is not a match due to having a different coloration from our specimen (blue-green with blue flash points, as opposed to yellow-green with red flash points for *H. v. viridiflavus*), and the fact that *H. v. destefanii* has a narrow distribution within the rift valley and not in the plains where this specimen was collected (Schiøtz 1999). Thus, we can confidently exclude this other subspecies as an identity for this specimen.

We also generated a mitogenome and constructed phylogenetic trees using BUSCO and 16S data in order to better understand the uniqueness of this genome compared to currently available full and partial genomes. In particular, *Hyperolius* frogs have structural variability in the mitogenomes of the few specimens already sequenced (Bittencourt-Silva et al. 2024), which has implications for mitochondrial-only based phylogenies, which we briefly explore.

## Methods & materials

### Sample collection and approvals

Our study was approved by the relevant Institutional Animal Care and Use Committee at Queens College and New York University School of Medicine (IACUC; Animal Welfare Assurance Number A32721–01 and laboratory animal protocol 19–0003). The frog specimen was sampled according to permits DA31/192/2010 and DA31/230/2010 provided by the Ethiopian Wildlife Conservation Authority. The specimen is housed at Zoological Natural History Museum of Addis Ababa University (ZNHM), Ethiopia awaiting catalogue (field number SB243). Liver tissue is stored in RNAlater at −80 °C at NYUAD.

### Whole genome sequencing

DNA extraction, sample and library preparations, as well as PacBio long-read whole genome sequencing and assembly were performed by Maryland Genomics at the Institute for Genome Sciences, University of Maryland School of Medicine.

#### Extraction

Approximately 25 milligrams of RNAlater-preserved frog liver tissue were used to extract DNA. The tissue was processed with the standard Dounce homogenizer protocol for tissue via the Circulomics Nanobind DNA Kit. In brief, the tissue was homogenized, lysed, and bound to the Nanobind disk to harvest genomic DNA. The resulting genomic DNA was assessed via the Femto Pulse instrument (Agilent, Santa Clara, CA) to confirm its integrity prior to library preparation.

#### Library preparation & sequencing

DNA samples were sheared on a MegaRuptor 2 (Diagenode, Denville, NJ) to an average size of 15 kb. Libraries were constructed using SMRTbell Prep Kit 3.0 (Pacific Biosciences, Menlo Park, CA) according to manufacturer's protocol. Adaptor-ligated libraries were size-selected on a BluePippin instrument (Sage Science, Beverly, MA) to remove fragments less than 9 kb in size. The size-selected libraries were bound to polymerase with the Sequel II Binding Kit 3.2, then sequenced with Sequel II Sequencing Kit 2.0 and SMRT Cell 8 M on a Sequel IIe instrument (Pacific Biosciences, Menlo Park, CA) for 30 h.

### Genome assembly

PacBio HiFi reads were used to perform de novo genome assembly using HiFiasm v0.16.1 (Cheng et al. 2021) with default parameters following the frog example in the HiFiasm github repository (https://github.com/chhylp123/hifiasm: hifiasm -o SB_243_Hyperolius_viridiflavus –primary -t 48 XLLUC_20220823_S64049_PL100262954-1_D01.ccs.fastq.gz XLLUC_20220909_S64411e_PL100262954-3_D01.ccs.fastq.gz XLLUC_20220929_S64411e_PL100262954-3_A01.ccs.fastq.gz. Assembly statistics including total assembly size, number of contigs, N50 and L50 were calculated using QUAST v5.0.2 (Gurevich et al. 2013). Two other genome assembly pipelines were also used: the Improved Phased Assembler (IPA) from PacBio software (v1.3.1; https://github.com/PacificBiosciences/pbipa) and Canu (Koren et al. 2017). These alternative assemblies were less continuous and had lower BUSCO scores and were not considered further.

#### Reference guided scaffolding

The *scaffold* function in *RagTag* (Alonge et al. 2022) was used to further scaffold the draft genome. Specifically, the recently published chromosome-level genome assembly of *H. riggenbachi* (NCBI: GCF_040937935; Vertebrate Genomes Project, https://vertebrategenomesproject.org/; Rhie et al. 2021) was used as a reference to assign contigs to potential chromosome matches. Gap size was inferred (option -r), and default values were used for all other parameters. The resulting pairwise mapping format output was further processed to estimate assembly completeness and continuity. Queries were filtered to include only those mapping to the chromosome scaffolds, and only the best hit per query sequence was kept (based first on the mapq then nmatch parameters). The GenomicRanges package v0.4.15 (Lawrence et al. 2013) was then used to calculate mapping coverage per 1mb windows, and the circlize package v1.50.2 (Gu et al. 2014) was then used to visualize both the mapping and coverage.

### Genome annotation

The evidence-based lift-over annotation software EviAnn v2.0.4 (https://github.com/alekseyzimin/EviAnn_release) was used to identify and annotate protein-coding gene and long non-coding RNA annotations. The transcripts of the genomes of *H. riggenbachi* (NCBI GCF_040937935) and *Xenopus tropicalis* (NCBI GCA_000004195, Hellsten et al. 2010) and all 1,969,996 Anura peptides from NCBI (downloaded on 02/10/2025) were used as reference transcripts and peptides, and EviAnn was run in lift-over mode with default settings. The two reference transcripts were chosen to balance evolutionary proximity in the *Hyperolius* dataset and the precision of the extensively curated *Xenopus* dataset, which include a well-assembled genome with chromosome-level scaffolds, rich functional annotations (GO terms, protein domains, expression data), and support from databases like Xenbase that include curated gene models and orthology relationships. These resources make it easier to transfer annotations accurately. The peptide set was expanded to include all Anura to obtain a larger reference set, as recommended by the software authors (Zimin et al. 2025).

### Mitogenome assembly and annotation

The complete mitogenome was extracted and assembled from the PacBio HIFI reads using MitoHIFI (Uliano-Silva et al. 2023) with the mitogenome of *Hyperolius marmoratus* (sp.) as a reference (NCBI NC_023381; Kurabayashi and Sumida 2013). We add the modifier (sp.) to the reference genome due to uncertainty in the exact taxonomic identity of this specimen as it was purchased in the pet trade and no photos or location of origin are known. Based on sequence similarity to *H. v. ferniquei* (NCBI AY603987), we are confident that this is a member of the *H. viridiflavus* superspecies group and a suitable comparison to our mitogenome. The *H. v. viridiflavus* mitogenome was then subsequently annotated using MitoFinder (Allio et al. 2020) and the mitogenomes of *Hydrophylax leptoglossa* (NCBI OR058745), *Pyxicephalus adspersus* (NCBI NC_044480), *Polypedates megacephalus* (NCBI OP965717; Cai et al. 2019), *Nidirana daunchina* (NCBI OR528757), *Mantella madagascariensis* (NC_007888; Kurabayashi et al. 2006), *Rana temporaria* (NCBI LR991693; Streicher 2021), *Rana coreana* (NCBI ON920705; Kim et al. 2023), *Rana kunyuensis* (NCBI KF840516; Li et al. 2016), *Odorrana ishikawae* (NCBI AB511282; Kurabayashi et al. 2010), and *Rana amurensis* (NCBI MF370348; Liu et al. 2017). The sequence was then inspected and circularized manually in Geneious Prime v. 2024.0.2 (https://www.geneious.com).

### Phylogenomic tree reconstruction

Single-copy BUSCO genes were extracted from 41 anuran and one caudate (*Pleurodeles waltls*) genome sequences available on NCBI using BUSCO v.5.8.3 with the tetrapoda_odb12 database (Simão et al. 2015; Manni et al. 2021). To select the genomes to include in the analysis, we focused on genomes of African species, included all genomes available for Afrobratrachia (n = 5 including partial, scaffold-level genomes), and added representants of other major anuran groups when available. We assembled a supermatrix using the BUSCO phylogenomics pipeline (available at https://github.com/jamiemcg/BUSCO_phylogenomics) and with all single-copied BUSCO genes present in at least 70% of the selected species, which amounted to a total of 416 genes. We then reconstructed a maximum likelihood phylogeny using IQ-TREE 2 (Minh et al. 2020) with 1,000 ultrafast bootstrap iterations, while simultaneously finding the best-fitting model for each partition using ModelFinder Plus (Kalyaanamoorthy et al. 2017) and merging partitions evolving under the same model (MFP + MERGE). Finally, we rooted the tree using *Pleurodeles waltl* as the outgroup and dated the tree using the *chronos* function in the R package *ape* (Paradis and Schliep 2019) and the following calibration points from Portik, et al. (2023): Crown-group age of Pelobatidae + Pelotidae: minimum = 86.3 Mya, crown-group age of Pipidae: minimum = 83.6 Mya, crown age of Myobatrachidae: minimum = 54.6 Mya, crown age of Bufonidae: minimum = 56.0 Mya, crown age of Ptychadenidae + Phrynobatrachidae: minimum = 24.5 Mya, crown age of Pyxicephalidae: minimum =39.5 Mya.

### 16S Phylogenetic tree reconstruction

The 16S sequence of *Hyperolius viridflavus viridiflavus* was extracted from the mitogenome previously assembled (see above). GenBank was searched for “*Hyperolius*” and “16S”, and relevant (n = 192) sequences were downloaded, as well as three 16S sequences from *Leptopelis karissimbensis* and *Leptopelis kivuensis*, to serve as outgroups. We aligned all sequences into a single alignment in Seaview v. 5.0.4 (Gouy et al. 2021) using the MUSCLE v5 algorithm (Edgar 2004), visually inspected and manually trimmed the sequences in the same software. We then reconstructed a maximum-likelihood tree with 100 bootstrap iterations using PhyML v. 3.1 (Guindon et al. 2010).

## Results and discussion

### Assembly

**Fig. 2.**
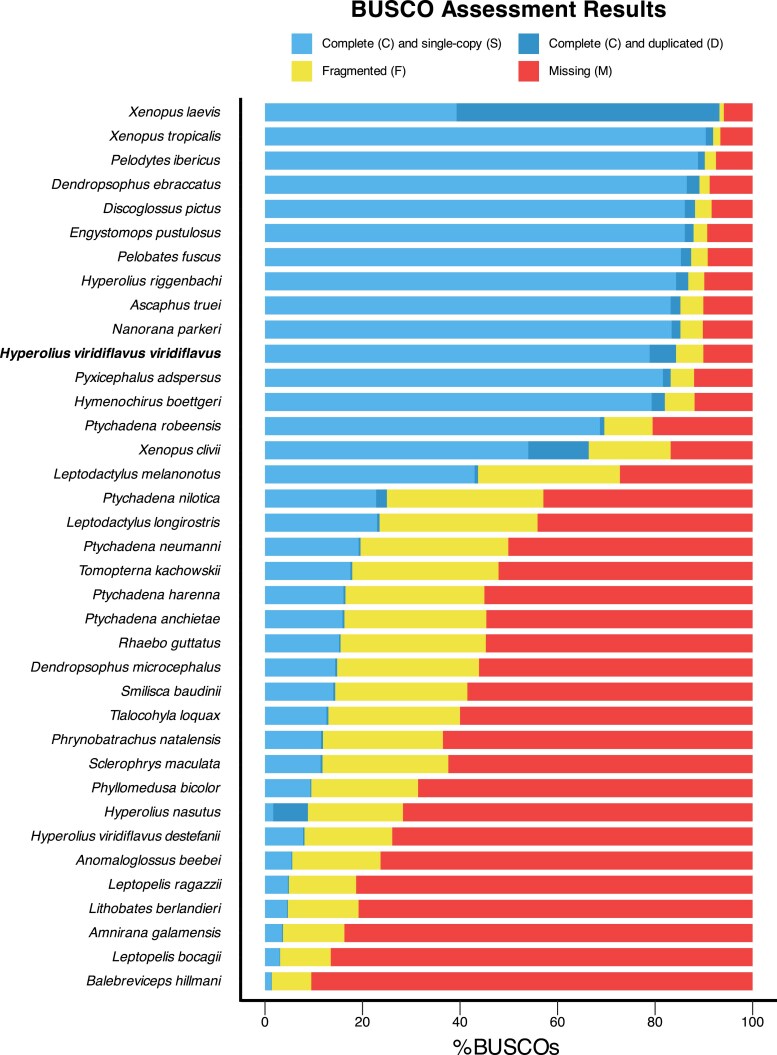
BUSCO completeness assessment across 37 anuran species obtained using BUSCO v.5.8.3 with the tetrapoda_odb12 database and sorted by completeness. Our new genome for *Hyperolius viridiflavus viridiflavus* is highlighted in bold. Photos from Sandra Goutte.

In total, approximately four terabytes of PacBio reads were generated. The assembled genome comprised 4,429,086,392 bp, spread across 10,009 contigs (all greater than 10,000 bp) with an N50 of 1,090,494 bp, and a GC content of 45.2%. The estimated genome size is ∼ 4.92 Gbp (https://www.genomeark.org/vgp-all/Hyperolius_riggenbachi.html). Genome completeness was assessed using BUSCO v.5.8.3 with the tetrapoda_odb12 database, which identified 84.2% of the 5,623 expected genes as complete (including 78.9% single-copy and 5.4% duplicated), 5.6% as fragmented, and 10.2% as missing (Fig. 2). The number of Ns per 100 kbp was 0. Although this draft genome is fragmented, it represents a major step forward in building whole genome resources for African frogs, which are underrepresented in online repositories. Compared to other anuran genomes within NCBI, our genome is among the most complete (Fig. 2). Our BUSCO scores are only slightly lower than the chromosome-level assembly of *Hyperolius riggenbachi*. This level of completeness is likely due to the use of long-read technologies, as many fragmented anuran genomes are short-read only (e.g. Illumina). The *Hyperolius riggenbachi* chromosome level genome (GCA_040937935) was not available in NCBI at the time of our *de novo* assembly creation (May, 2023), nor was the partial genome of *H. viridiflavus destefanii* (∼2Gb, or less than 50% of expected size; GCA_046244975). Thus, these genomes were used only in post-assembly analyses.

### Scaffolding

**Fig. 3.**
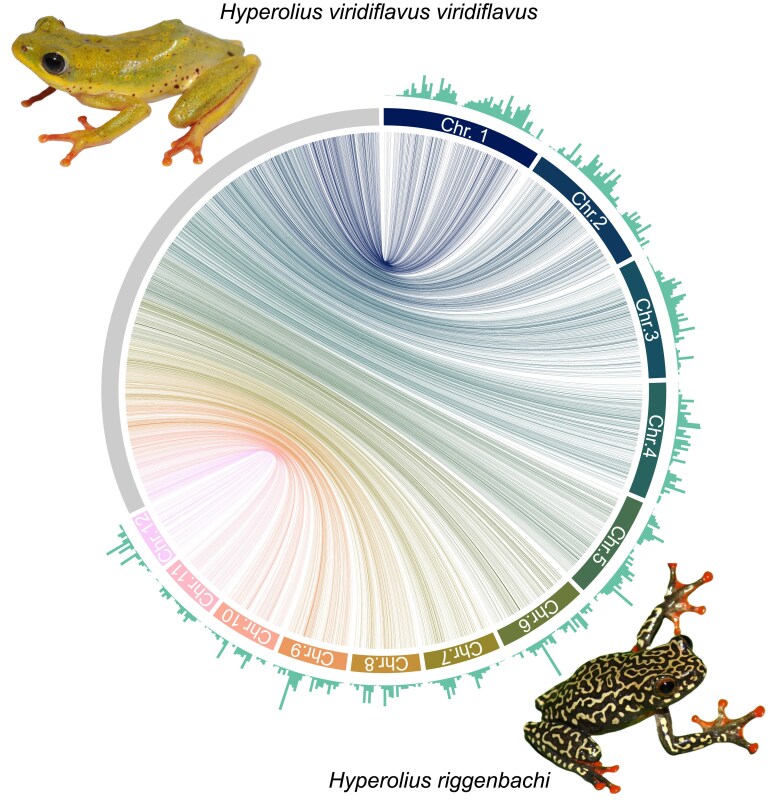
Circos plot of scaffolding results. Colored segments represent the 12 assembled chromosomes of *H. riggenbachii* with the gray segment representing all *H. viridiflavus* contigs mapping to these reference chromosomes. Connecting ribbons show the best mapping result. Bar charts per reference chromosome represent relative mapping coverage per 1mb windows. Photos from Sandra Goutte.

RagTag could map 7,931 *H. viridiflavus* contigs (4,256,193,765 bp) to the *H. riggenbachii* chromosomes while 2,078 contigs (172,892,627 bp) were unmapped and 9,953 gap sequences (61,762,072 bp) were inferred, resulting in an average per base coverage of 0.0654 (SD = 0.0666) across 1mb windows (Fig. 3). Noticeable dips in mapping coverage were observed at the ends and mid-sections of chromosomes, which likely harbor highly repetitive sequences.

Despite the nearly identical size of this genome to the *H. riggenbachi* chromosome-level genome (∼4.9 Gbp), the average coverage was low when comparing the two. As our BUSCO scores were generally good, we believe that most differences are in the non-coding regions and repeats. We also anticipate that there may be misassembled contigs and potentially multiple haplotigs in the assembly which could map to the same region of the *H. riggenbachi* genome due to the highly repetitive nature of frog genomes (sometimes exceeding 70%; Lv et al. 2024). Future studies, including incorporating looping data, will help resolve full chromosomal assembly for *H. viridiflavus viridiflavus*.

### Gene annotation

The EviAnn annotation pipeline recovered 42,930 transcripts and 29,809 genes, of which 27,983are protein coding in the *H. viridiflavus* assembly (Table 1). In addition, the pipeline annotated 942 long non-coding RNAs and 918 pseudo genes and a total of 39,549 distinct proteins. This is comparable to the average of 24,039 protein-coding genes (standard deviation = 10,894.6) identified across the 32 amphibian reference-level genomes currently accessioned on GenBank. The proteomes of amphibians are still extensively unknown, with the UniProt proteome database containing only 9 amphibian representatives, whose protein numbers range from 25,860 to 61,882 proteins.

**Table 1.** Summary of genome annotation for *H. viridiflavus viridiflavus*.

Number of genes	29,809
Number of protein coding genes	27,983
Number of processed pseudo gene transcripts	918
Number of processed pseudo genes	918
Number of transcripts	42,930
Number of long non-coding RNAs	942
Number of distinct proteins	39,549

### Mitogenome

**Fig. 4.**
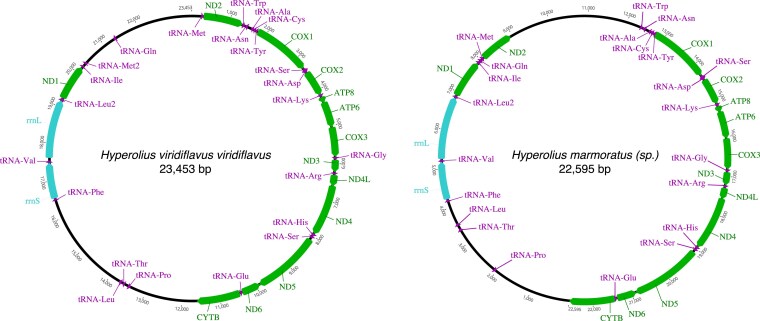
Comparison of the mitogenomes of *Hyperolius viridiflavus viridiflavus* and closely related *Hyperolius marmoratus* (*sp.*). green: protein-coding genes; blue: ribosomal genes; purple: transfer RNA genes. Photos from Sandra Goutte.

The mitogenome is 23,453 bp in length, and we retrieved 13 protein-coding genes, 23 tRNAs, and 2 rRNAs. Nucleotide composition is A: 33.8%, C: 21.1%, G: 11.7%, T: 33.4%. The gene order is the same as in the mitogenome of closely related *Hyperolius marmoratus* (another member of the *Hyperolius viridiflavus* superspecies group), although the inter-gene regions between ND1 and ND2 on one hand, and ND2 and COX1 on the other hand differ in length (Fig. 4). The mitogenome of *H. viridiflavus viridiflavus* also differs from that of *H. marmoratus* by a second copy of the transfer RNA for Methionine (tRNA-Met) between ND1 and ND2 genes. Variation in tRNAs (e.g. *trnL*, *trnT*, *trnP*) and genes (e.g. pseudogene of ND2) could be common in *Hyperolius* frogs (Kurabayashi and Sumida 2013), with *H. substriatus* showing a repeat of the “CR + tRNA LTPF cluster’ in the WANCY region (Bittencourt-Silva et al. 2024), and *H. marmoratus* possessing a region with pseudogenes of ND2, trnM, and D-loop control region and regions labeled as “noncoding region for possible remnant of a genomic duplication” (Kurabayashi and Sumida 2013). Future sequencing of whole mitochondrial genomes across Anura will help clarify the level of duplications and rearrangements that have taken place.

### Phylogenetic placement BUSCO

**Fig. 5.**
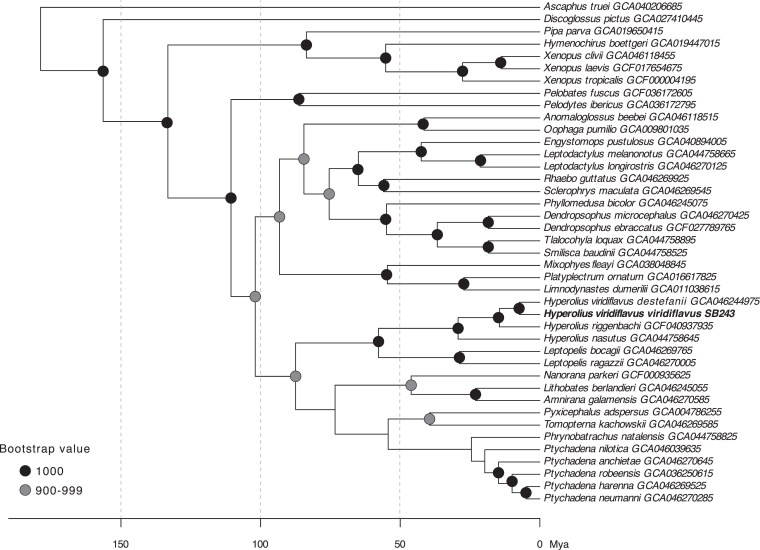
Dated Maximum likelihood phylogeny based on 416 single-copy BUSCO genes. Outgroup of *Pleurodeles walt* removed. GenBank assembly numbers are given at the tips after the species names. Ultrafast bootstrap support values (n = 1,000 iterations) are given at the nodes as shaded circles, with unlabeled nodes representing bootstrap values below 700. Photos from Sandra Goutte.

Our new sequence is similar but distinct from a “*H. viridiflavus*” sample (voucher TJC476, museum accession number CAS253644, NCBI GCA_046244975) identified as *H. v. destefanii* (BioSample SAMN10907069; Portik et al. 2019). This identification is confirmed by collection locality (near lake Awasa; 7°05'54.4”N 38°24'59.0”E), coloration, and communication with NCBI submitting author T. Colston. Genetic distance is greater between these two subspecies (*H. v. viridiflavus* and *H. v. destefanii*) than between other named full sister species pairs in this phylogeny (e.g. *Ptychadena harenna* and *P. neumanni*) (Fig. 5).

### 16S Phylogenetic tree reconstruction

**Fig. 6.**
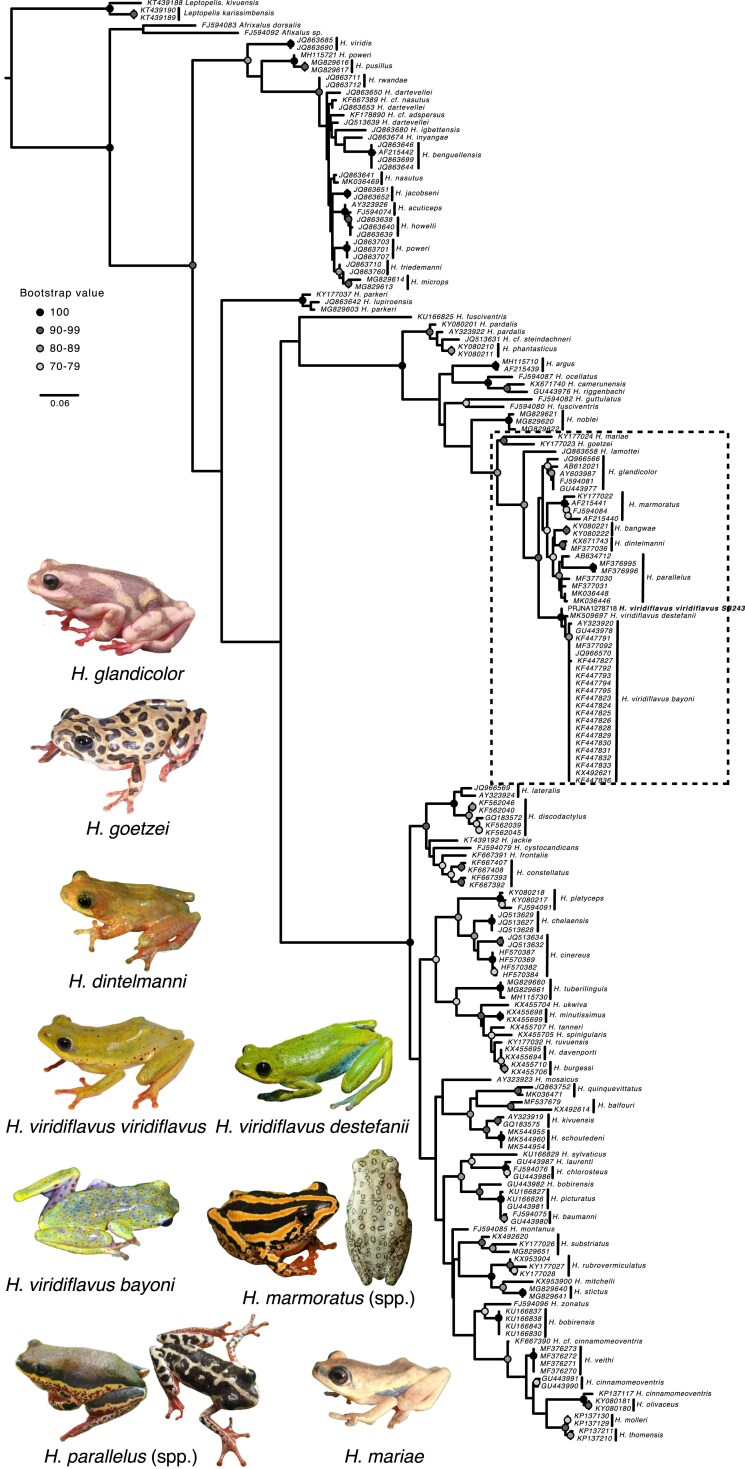
Maximum likelihood phylogeny based on mitochondrial 16S rRNA sequences. Bootstrap support values (n = 100 iterations) are given at the nodes as shaded circles, with unlabeled nodes representing bootstrap values below 70. Branch lengths represent genetic distance. GenBank accession numbers for the 16S sequences used are given at the tips along with the species names. Our genome (field ID SB243), is bold and is referenced by its NCBI BioProject ID. The *Hyperolius viridiflavus* superspecies complex is outlined in a dashed box, and representative photos (when available) are included. Photos from Tim Colston, Sandra Goutte, Lucinda P. Lawson, H. Christoph Liedtke, Daniel M. Portik, and iNaturalist photos (taking into consideration location and coloration for each taxon) from Colin Ralston, Wynand Uys, Lorenzo Vinciguerra, and chemp used under CC BY-NC 4.0 creative content non-commercial license where they have not been edited except by removing the backgrounds. Photos from Sandra Goutte.

Our mitochondrial 16S phylogenetic tree (Fig. 6) is similar in topology to recent *Hyperolius* phylogenetic trees (Portik et al. 2023). 16S is a highly variable sequence and is particularly effective at resolving close relationships (e.g. sister species), but substitution rates are easily saturated leaving older nodes poorly resolved. We find these same results, with high confidence in recent divergences near the tips (dark colored circles) but low confidence in deeper divergences (below 70% bootstrap confidence on most basal nodes). The closest relatives to *H. viridiflavus viridiflavus* are the *H. viridiflavus destefanii* specimen and specimens from Uganda, Kenya, and Rwanda and identified as *H. viridiflavus bayoni*. Due to the spatial proximity of these subspecies, further investigations into the uniqueness of these lineages may be warranted. *Hyperolius* species are known to have mito-nuclear conflict in their phylogenetic signals (Bwong et al. 2020; Lawson et al. 2025; Nečas et al. 2025), and interpretations based solely on 16S sequences are not advised. Additionally, we note that as mentioned in Channing (2022), certain 16S sequences were misidentified on NCBI, and we crossed information from the available metadata, linked literature, and used BLAST to assign a corrected identification. For example, the sequences FJ594083 and FJ594092 identified on NCBI as *H. lamottei* and *H. sylvaticus*, respectively, were found to belong to the genus *Afrixalus.*

### Summary

Here, we provide the first whole-genome assembly of a member of the *Hyperolius viridiflavus* superspecies complex based on long-read technology. Long read technology is vital for amphibian genomes which have high repeats (can exceed 70%) and marks a major advance over short-read only genomes which appear to be able to only capture ∼50% of the genome (e.g. *H. viridiflavus destefanii* partial genome in NCBI). The genome of *H. viridiflavus viridiflavus* will serve as an invaluable resource for studying the genetics, biology, and evolution of Afrobatrachids, and will also aid in enhancing conservation strategies for this sub-Saharan African superspecies complex. Genomes of amphibians have proven to be difficult to assemble for a range of factors including large genome sizes and high repetitiveness (Kosch et al. 2025), but long-read sequencing strategies and increased representation of taxa across the diversity of amphibians enable a more complete understanding of genome diversity in this poorly understood group. Though this *Hyperolius viridiflavus viridiflavus* genome is fragmented, it serves as a resource for future work in the ∼400 species of Afrobatrachia (sensu Frost et al. 2006). This clade (Families: Arthroleptidae, Brevicipitidae, Hemisotidae, Hyperoliidae) represents over half of the total frog species diversity in sub-Saharan Africa (Frost 2020). As of this writing, only *Hyperolius riggenbachi* has a chromosome level genome available on NCBI out of all Afrobatrachia, and no other genomes in NCBI (scaffold-level) have over half of the genome sampled based on expected size (∼4.3 Gbp). This addition increases the available resources in this under-studied clade.

## Data Availability

The assembled genome has been deposited at NCBI GenBank under the genome BioProject ID PRJNA1278718. Genomic raw reads are available at NCBI SRA under the accession numbers SRR34793120, SRR34793119, SRR34783217, SRR34783218. BioSample accession SAMN49206974. Annotations (GFF file from EviAnn annotations and RagTag mapping file) are available on NCBI with the genome and FigShare (https://doi.org/10.25387/g3.29821394).
